# Supplementary material for: Minerva: an alignment- and reference-free approach to deconvolve Linked-Reads for metagenomics
Source: Genome Res. 2019 Jan;29(1):116–24. doi: 10.1101/gr.235499.118 (PMC6314158; doi:10.1101/gr.235499.118)
Supplement: Supplemental Material [file supp_29_1_116__index.html]

Minerva: an alignment- and reference-free approach to deconvolve Linked-Reads for metagenomics — Supplemental Material 

# Minerva: an alignment- and reference-free approach to deconvolve Linked-Reads for metagenomics

## Supplemental Material

- supplemental\_materials\_IH.zip
- Supplemental\_Fig\_S3.jpg
- Supplemental\_Fig\_S1.jpg
- Supplemental\_Fig\_S2.jpg
- Supplemental\_Code.zip
- Supplemental\_Material.docx
