# Supplementary material for: Minerva: an alignment- and reference-free approach to deconvolve Linked-Reads for metagenomics
Source: Genome Res. 2019 Jan;29(1):116–24. doi: 10.1101/gr.235499.118 (PMC6314158; doi:10.1101/gr.235499.118)
Supplement: Supplemental Material [file supp_gr.235499.118_supplemental_materials_IH.zip › supplemental_materials_IH/minerva_supplement_IH.docx]

**Minerva Supplementary Materials**

**Read Clouds**


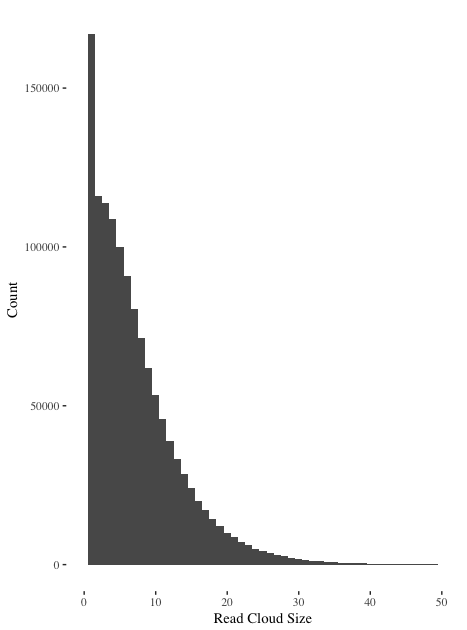


We show a histogram of observed Read Cloud sizes in this figure. This histogram is truncated and does not show a long tail of large Read Clouds. This figure shows that the majority of Read Clouds are small.


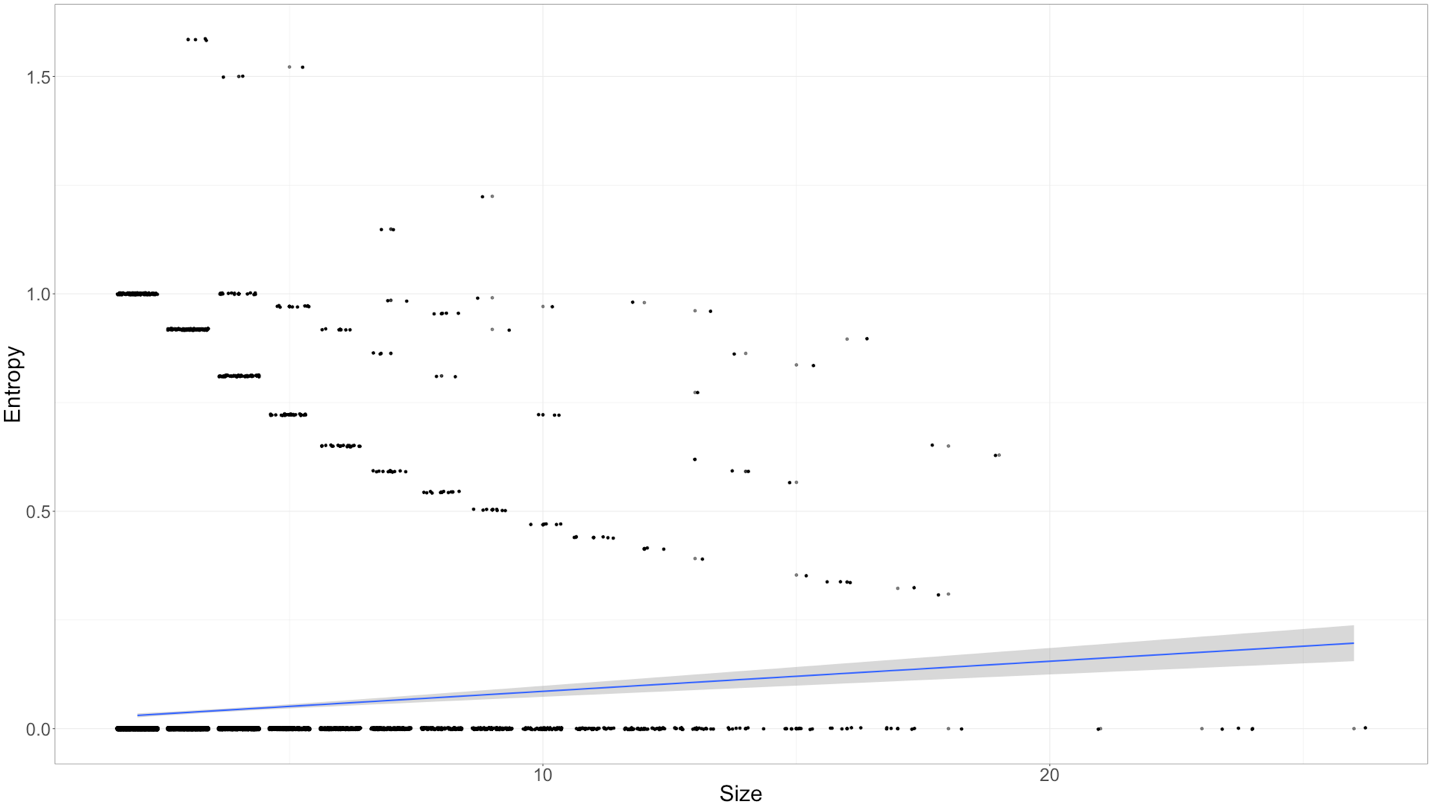


We show a scatter plot of enhanced Read Cloud size versus entropy. Entropy only increases slightly with enhanced Read Cloud size.

**Effect of Parameters**


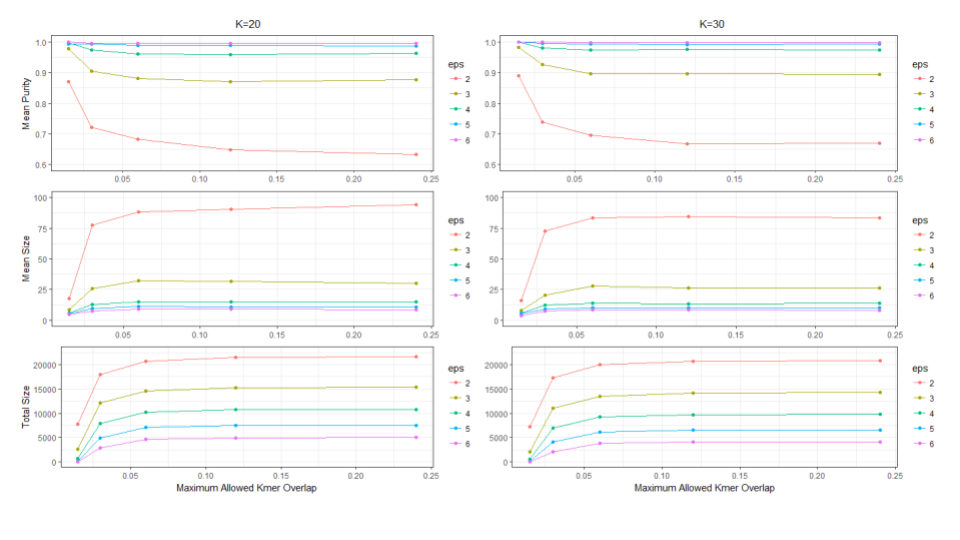


We summarize the impact of various parameters on model performance in this figure.

**Enhancements to our Algorithm**

Depending on the parameters and dataset used the clustering step may produce clusters that are large and imprecise or small and incomplete; this is partly a consequence of using a small number of discrete components to define connections. To mitigate this issue we have introduced two enhancements to the clustering step that may optionally be used. We have based these enhancements on the two step procedure used for community recovery by Abbe (2016) and Mossel, (2014).

We term the first enhancement read-rescue; in some instances it is useful to rescue components that consist of single reads by connecting them to clusters that they unambiguously share connections with. To do this all single reads are checked for connections to any read in a cluster. If the single read is connected to enough reads (by a user supplied parameter) in no more than one cluster the read is added to that cluster. We term the second enhancement edge-cracking; when clusters are too large it is useful to break certain edges that bridge two individually well-connected components. To do this all edges in the adjacency matrix are considered. If the two reads attached to an edge do not share enough neighboring reads (by a user supplied parameter) the edge is removed from the adjacency graph.

**Failed Models**

While developing this model we tested several variants that performed poorly. In this section we report these models as negative results, parameters and outcomes may be found in the supplement. We define three distinct components of our model:

- Barcode reduction, a technique to generate a matrix with rows representing reads and columns related to barcodes
- Cell weighting, a technique to reweight the cells of our reduced matrix
- Clustering, alternate techniques to cluster our reduced, reweighted matrix

We tried several techniques to reduce our matrix (our final technique was to build a bipartite graph that was converted into an adjacency matrix) including: Principal Component Analysis, Latent Dirichlet Allocation (treating each read as a document of barcodes), the jaccard, dice and kulsinski distance matrices between reads.

For most reductions we tried to reweight our matrices by normalizing row and column sums. We also tried term frequency - inverse document frequency and just inverse document frequency. For our final model we did not use any reweighting scheme.

To cluster our reduced and reweighted matrix we tried xmeans, kmedoids, hierarchical clustering with various linkages and Markov clustering. Hierarchical clustering yielded reasonable performance but dbscan was faster, had more intuitive parameters, and had slightly better performance.

Additionally, we tried to use LDA globally (treating each barcode as a document of kmers) to discover sets of kmers that uniquely identified fragments. This technique provided surprisingly good barcode deconvolution (and automatically defined globally clusterings of barcodes) but was extremely computationally costly and did not yield results as good as our final model.
