# Supplementary figures and images for: Minerva: an alignment- and reference-free approach to deconvolve Linked-Reads for metagenomics
Source: Genome Res. 2019 Jan;29(1):116–24. doi: 10.1101/gr.235499.118 (PMC6314158; doi:10.1101/gr.235499.118)

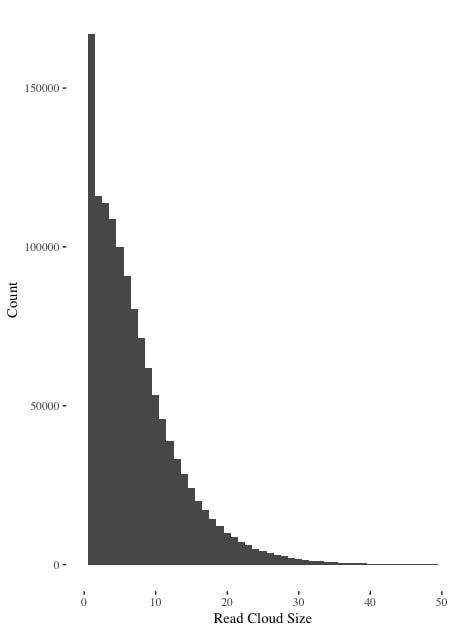

Supplement: Supplemental Material [file supp_gr.235499.118_supplemental_materials_IH.zip › supplemental_materials_IH/Supplemental_Fig_S1.jpg]

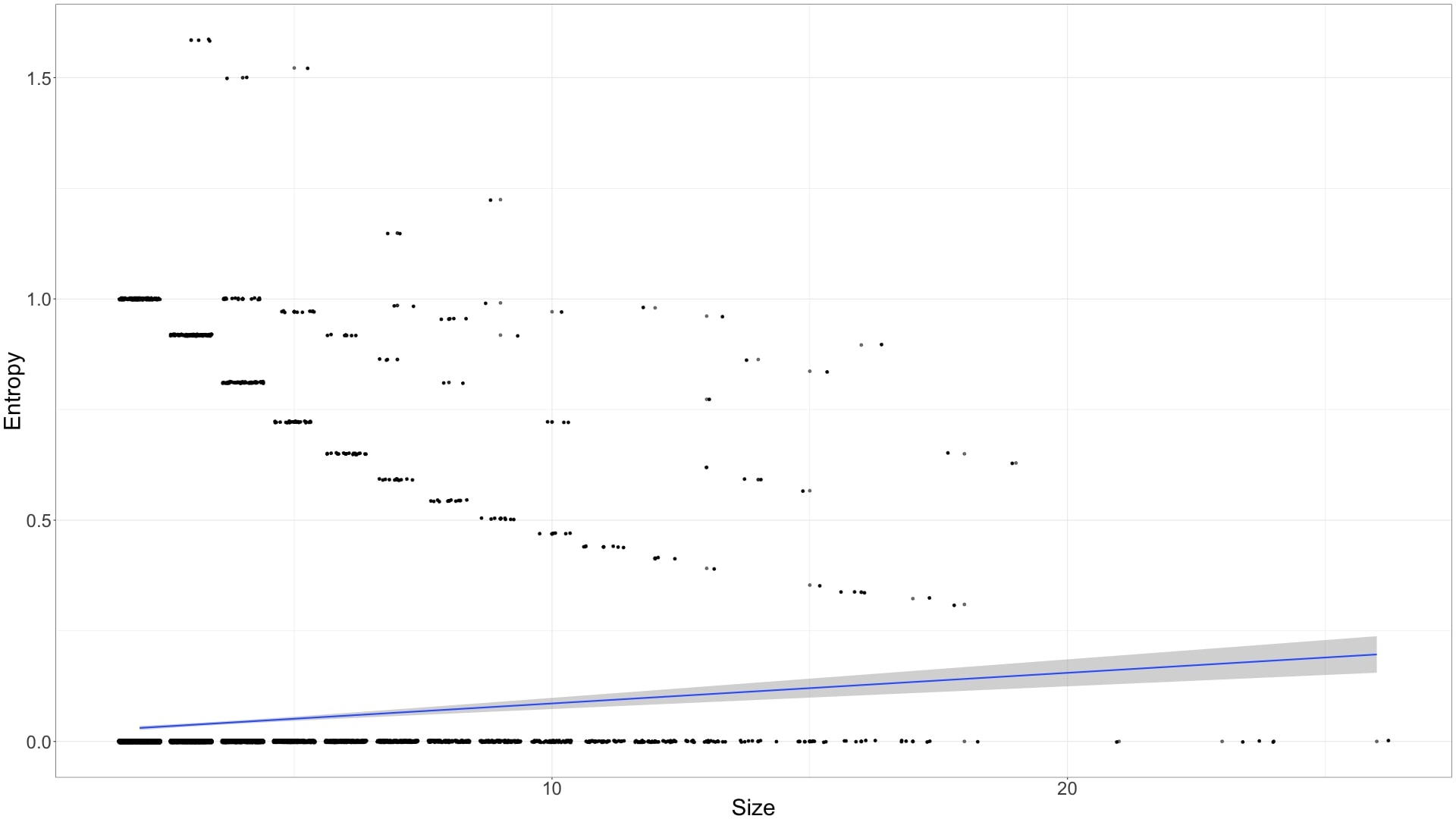

Supplement: Supplemental Material [file supp_gr.235499.118_supplemental_materials_IH.zip › supplemental_materials_IH/Supplemental_Fig_S2.jpg]

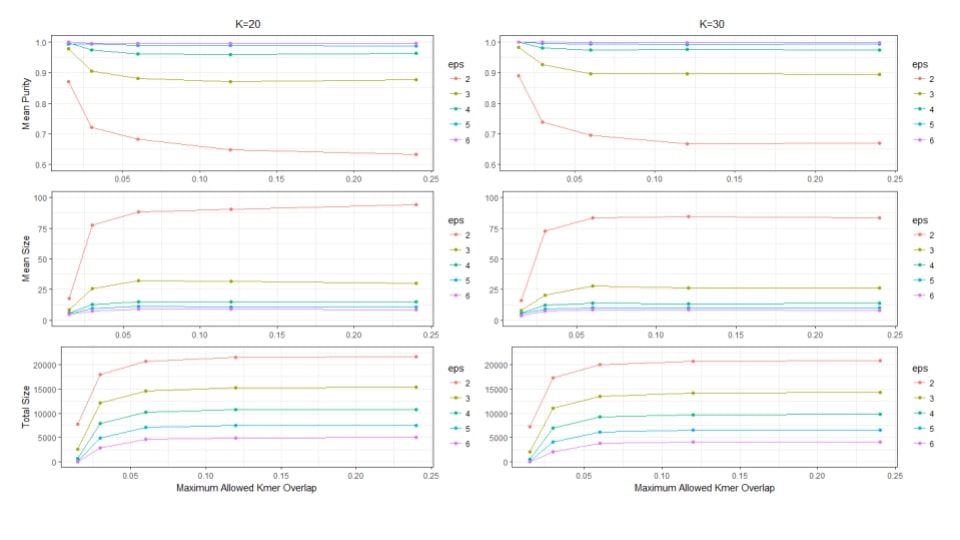

Supplement: Supplemental Material [file supp_gr.235499.118_supplemental_materials_IH.zip › supplemental_materials_IH/Supplemental_Fig_S3.jpg]
