# Supplementary material for: Minerva: an alignment- and reference-free approach to deconvolve Linked-Reads for metagenomics
Source: Genome Res. 2019 Jan;29(1):116–24. doi: 10.1101/gr.235499.118 (PMC6314158; doi:10.1101/gr.235499.118)
Supplement: Supplemental Material [file supp_gr.235499.118_supplemental_materials_IH.zip › supplemental_materials_IH/response_to_editor_IH.docx]

On Fri, Nov 16, 2018 at 6:01 PM [genomere@cshlbp.org](mailto:genomere@cshlbp.org) <[genomere@cshlbp.org](mailto:genomere@cshlbp.org)> wrote:

MS ID#: GENOME/2018/235499
MS TITLE: Minerva: An Alignment and Reference Free Approach to Deconvolve
Linked-Reads for Metagenomics

Dear Dr. Hajirasouliha,

We are returning this new submission for the following reasons:

*Please have all data at BioProject under accession number PRJNA505182 released at
this time.*

Done

*Please add pointers to Fig. 3 part 1 and Table 2.*

Done

*Please upload individual high resolution image source files (in acceptable format,
not png) for both main text figures and Supplemental Figures (which should include
legends).*

We uploaded all figures (main and supplemental) in JPG format.

*Please upload Minerva source code to the Supplemental Material for posterity, point
to it there from the Data access section as well as in GitHub. Please rewrite this
section in our boilerplate language.*

Done

*Relocate the following information from references to where it is mentioned in the text: 
"10x genomics long ranger pipelines:*
[*https://urldefense.proofpoint.com/v2/url?u=https-3A__support.10xgenomics.com_genome-2Dexome_software_pipelines_latest_advanced_other-2Dpipelines&d=DwIFaQ&c=lb62iw4YL4RFalcE2hQUQealT9-RXrryqt9KZX2qu2s&r=lLpNs9kSLxNilzstznvQuhgD3z2uqln9PYyN8Nz2V74&m=yEpyySufYRCIZ08htn022LMZTjQWYW1LyRGt9qMU3lY&s=nogsIOl9AmvV-kz-YtWRUuv8NnqwVojWsZfWTKqsUrA&e=*](https://urldefense.proofpoint.com/v2/url?u=https-3A__support.10xgenomics.com_genome-2Dexome_software_pipelines_latest_advanced_other-2Dpipelines&d=DwIFaQ&c=lb62iw4YL4RFalcE2hQUQealT9-RXrryqt9KZX2qu2s&r=lLpNs9kSLxNilzstznvQuhgD3z2uqln9PYyN8Nz2V74&m=yEpyySufYRCIZ08htn022LMZTjQWYW1LyRGt9qMU3lY&s=nogsIOl9AmvV-kz-YtWRUuv8NnqwVojWsZfWTKqsUrA&e=)*."*

Done

*Please reformat all references, as they are not in this journal's format. Please see
the instructions to authors for proper format.*

Done

*Please number Supplemental figures and add pointers to them individually from the
main text.*

Done

*Please convert all x's (and *'s) to multiplication symbols where you mean coverage,
concentration, or "times" (e.g., 3*106 barcodes on page 4).*

Done

*Once again, please remove section/subsection numbering, as well as pointers to
subsections throughout.  If necessary, please point to subsection names within the
text, rather than numbers.

Please remove all subsections (numbers and headings) in the Introduction.*

Done

*Regards,

Genome Research*

We thank the editor and reviewers for the positive decision and insightful feedback. Below please find our responses to the minor comments.

*Please reduce abstract to 250 words or fewer.* DONE

*Please remove bolding from Minerva.* DONE

*Please remove subsections in the Introduction, and section/subsection numbering, as*

*well as pointers to subsections throughout.*

We ask the editor to consider us allowing to keep subsections in the introduction as we believe they improve the legibility of the paper.

*Redo figure 1 and reference from text (reviewer 1 & 2)*

*Please provide pointer to Figure 1.*

We have removed figure 1 and instead added a reference to the original paper describing the linked-read sequencing technique with a much more detailed explanation of linked reads.

*Please remove Glossary and define each term at first mention.* DONE

*Please correct:* DONE

*10X Genomics —>  10x Genomics*

*Hiseq —>  HiSeq*

*kmers —>  k-mers (k should be in italics)*

*Please convert all x's (and *'s) to multiplication symbols where you mean coverage,*

*concentration, or "times".* DONE

*Table 4 label is Table 3?* DONE

*Please ensure that all sequence data (Datasets 1 and 2) have been deposited in an*

*appropriate public database. Accession numbers should be included in the Data access*

*section.*

We have included sequence data in our supplementary materials and continue to provide it on our site.

*Please upload Minerva source code to the Supplemental Material for posterity, point*

*to it there from the Data access section as well as in GitHub. Please rewrite this*

*section in our boilerplate language.* DONE

*Please reformat all citations and references, as they are not in this journal's*

*format. Please see the instructions to authors for proper format.* DONE

*References 9 and 13 are published, please check all preprint references and update*

*when published.* DONE

*Please provide citation for the 10x Metagenomics Consortium (Pg 5).*

No reference for the consortium exists. In lieu of a reference we have detailed that the consortium is a small group of PIs in the acknowledgement section. We removed the term 10x Metagenomics Consortium from the main text.

*Supplemental Figures should be renumbered per our Supplemental File naming guidelines*

*below. Further, is Supplemental Figure 1, in the Supplemental Material (section 2.4),*

*Figure 6?*

*Supplemental Material should be uploaded as Supplemental (not source) files.*

*Please include Supplemental Figure and Table legends with their respective*

*Supplemental Figures and Tables in a well-organized Supplemental Material document,*

*and upload as a Supplemental file(s), not as source files. Please also ensure*

*Supplemental files themselves are named according to our specific convention, with*

*underscores connecting all segments in each file name and with the file-type*

*extension in view: Supplemental_Fig_S1.pdf, Supplemental_Fig_S2.eps,*

*Supplemental_Table_S1.xlsx, Supplemental_Table_S2.docx, Supplemental_Movie_S1.mov,*

*Supplemental_Methods.pdf, etc.  Each supplemental figure, table, movie, or data file*

*must be cited in the main text; citations and supplemental file names must match*

*(e.g., Supplemental Fig S1 in text points to Supplemental_Fig_S1.pdf, etc.).* DONE

*Flip Table 2 so dataset 2 only follows dataset 1 (reviewer 1)* DONE

*Confirm bcs are added to 3’ end of 5’ read (reviewer 2)* DONE

Please note that the document in question (CG00044_10x_Techical_Note_LinkedReads.pdf) shows a cartoon where barcodes are attached to the left side of DNA molecules but does not include any reference to the orientation of the molecules in the cartoon. Other materials from 10x Genomics make clear that the 10x barcode (aka gemcode) is ligated to the 3’ end of read 1. All Linked-Read raw data sets support this as well.

*Note total number of barcodes (reviewer 2)* DONE

Added order of magnitude to number of 3’ barcodes.

We changed the exact text to the more clear: ‘We note that our solution requires information from multiple 3' barcodes but that this is not necessary to state the barcode deconvolution problem generally’

*Change justification for real communities (reviewer 2)* DONE

*Check table numbers and figure legends (reviewer 2)* DONE

*4. Section 1.4: "For a set of Read Clouds a solution to the barcode deconvolution problem would be the set of solutions for each individual Read Cloud." I mentioned in my previous comments that this isn't obvious to me. Clarify.* DONE

We have attempted to clarify more by making our language more mathematically precise: ‘A solution to the barcode deconvolution problem for a set of Read Clouds would be a map from each Read Cloud to a function which solves the barcode deconvolution problem for that Read Cloud.’

*Trivial parallelization and separate barcodes. (reviewer 2)* DONE

We have clarified that the algorithm is trivially parallelizable across 3’ barcodes. Essentially the barcode deconvolution problem can be solved for individual barcodes in parallel as each barcode can be treated independently.

*8. Section 2.5: The section that starts "Unlike our graph based algorithm this model had a large number of parameters that could be adjusted until a model that fit the data well was found..." I had not mentioned this in my previous review, but this sounds like overfitting to me and left me confused about what the authors did. Clarify.*

*9. Section 2.5: "A key advantage of LDA is that it can classify words based on context (i.e. a river bank vs. a financial bank), this may be useful for classifying conserved motifs." Earlier in the paragraph you say that "LDA does not consider what order words in a document occur". Seems contradictory?*

We submit that both these statements relate to Topic Modeling and LDA and the discussion of why they are correct is out of scope for this paper. However we removed the reference to ‘our graphical model’ since it is out of place and not relevant.

We protest that is not at all contradictory to say that LDA uses context and that it ignores word order, word order is a special, limited, case of context. However, we have changed ‘classify word’s to ‘distinguish synonyms’.

*11. Figure 4 (which was figure 2) has 5 labeled parts but 6 steps in the legend that only sort of match the 6 steps in the following text.*

*The first step in their algorithm is described:*

** in the figure - "We obtain reads with the same barcode grouped into a read-cloud"*

** in the figure legend - "Fragments are generated"*

** in the following manuscript text - "Read Clouds are parsed, Read Clouds below a certain size (dropout) are dropped"*

*When referring to figure 4:*

*4.2.2 The Model*

*"Initially each Read Cloud in a given dataset is parsed into a set of minimizing kmers (see section 4.4, figure 4 part 2)."*

*Which part 2? The part in the figure, the legend or the text because they are all different.*

*I was pretty clear in my previous comments that, if you are going to refer to numbered "steps" in a pipeline figure, you need to be clear, consistent and use number labels.*

We left an extra step (step 1) in the caption for figure 4 based on an old version of the caption. With that step removed from the caption all references between the text and the figure align. We have significantly edited the figure caption to make this more clear.

We never assert any specific correspondence between the figure and our explicit list of the algorithmic steps even though the only meaningful difference is the inclusion of step 1 in the text: ‘1. Read Clouds are parsed, Read Clouds below a certain size (dropout) are dropped’. This step is abstract and we have updated our figure to make it clear that it only represents the process for a single barcode. We feel justified including more detail in the text than in the figure.

*Section 1.4: The problem statement is improved, but the notation is still confusing a few places. The letter “f” is first defined as the number of fragments (an unknown constant) and then used with a subscript to denote a fragment in the vector F (an unobserved random variable). Also, the largest subscript in F is n, which is the same as the largest subscript in R. For the problem to be tractable and realistic, you need the number of fragments to be less than the number of reads (i.e., more than one read per fragment). While n is certainly the maximum possible number of fragments, it is confusing to use n when in real data the number of fragments is smaller. How about using “m” rather than “f” for the number of fragments and having the largest subscript in F be m? You could then state that m is less than or equal to n.* DONE

We agree and have updated our notation accordingly. The set of all fragment classes (F-vec) is the inclusive range from 1 to *f* where *f* properly represents the total number of fragments.

*Add genome sizes and rel abund for species in each dataset (reviewer 3)* DONE

*Mention more complex communities in discussion (reviewer 3)* DONE

*Larger figures (reviewer 3)* DONE

*Section 4.1:*

*-	It would be useful to introduce the fragments F_i as a vector of F_i,j or to simply use the F_i,j notation from the start. The notation F_i (without being clear that this is a vector) masks the fact that there are multiple fragments.*

We have clarified vectors and the purpose of indexing variables.

*-	In the equation for P(G_w = G_y), explain the assumption behind taking the limit as the number of genomes goes to infinity? How does this mesh with your using communities with five or eight species?*

We protest that our mathematical model is not based on communities with only a small number of species however we have added a note ‘In real communities this is an approximation that improves as the total number of species increases.’

*-	The phrases “without a given genome” and “without given genomes” sound a bit odd, as if the fragments do not come from a genome (which might be misconstrued as referring to contaminant fragments). I follow that you mean without conditioning on a specific genome or pair of genomes. But it might be better to say “without conditioning on a given genome” or “without conditioning on their genome sources”, or something similar.*

We agree this change will reduce confusion and have duly included.
